# Cutaneous infection caused by *Mycobacterium chelonae* in an immunocompromised individual

**DOI:** 10.1590/0037-8682-0514-2025

**Published:** 2026-03-02

**Authors:** Lucas Benício dos Santos, Rafael Henrique Bento Elizeu, Lida Jouca de Assis Figueredo, Élida Aparecida Leal, Silvana Spíndola de Miranda

**Affiliations:** 1 Universidade Federal de Minas Gerais, Faculdade de Medicina, Departamento de Propedêutica Complementar, Belo Horizonte, MG, Brasil.; 2 Universidade Federal de Minas Gerais, Faculdade de Medicina, Departamento de Clínica Médica, Belo Horizonte, MG, Brasil.; 3 Fundação Ezequiel Dias, Serviço de Doenças Bacterianas e Fúngicas, Belo Horizonte, MG, Brasil.

**FIGURE 1:**
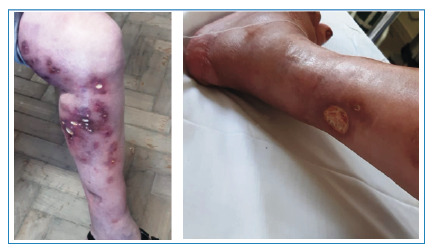
Numerous purplish hyperchromic macules, associated with hyperemia and edema, and disseminated ulcers on the left lower limb. Circular ulcers, with well-defined edges and a base filled with purulent exudate. 2021, at the time of diagnosis.

**FIGURE 2:**
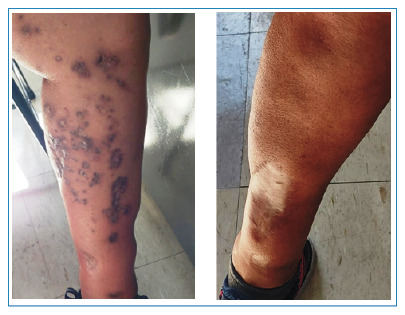
Lower left limb showing the healing of previous lesions associated with hyperpigmented hyperchromic macules, some with dry crust formation. Anterior region of the lower right limb, showing a flat scar with well-defined smooth edges. 2023, eighteen months after treatment.

A 55-year-old woman with type II diabetes, a former smoker, and a history of immunosuppression was treated with methotrexate for pyoderma gangrenosum between 2009 and 2013. During this period, the lesions exhibited intermittent improvement followed by worsening. In 2021, she was referred to the Tuberculosis Reference Clinic for a skin biopsy with positive bacilloscopy and a positive culture for rapidly growing mycobacteria. At that time, hyperchromic, hyperemic, and edematous macules and circular ulcers with purulent exudates that drained spontaneously were observed on her lower limbs (Figure 1). The identification tests confirmed the presence of *Mycobacterium chelonae* ^1^. The treatment lasted 18 months and initially included amikacin, clarithromycin, and moxifloxacin^2^. Sensitivity testing showed resistance to moxifloxacin, which was replaced with clofazimine. In this moment, bacilloscopy and mycobacterial cultures were performed and the wounds tested negative for draining secretions. By the end of the treatment, the lesions were reduced, which were accompanied by hyperchromic macules, some of which had dry crusts (Figure 2).

Nontuberculous mycobacteria are ubiquitous and can cause diverse conditions, from asymptomatic colonization to infections^2^ ^,^ ^3^. Diseases caused by *M. chelonae* are often associated with invasive procedures^4^. In this case, the microorganism was considered opportunistic and favored by prior immunosuppression^1^ ^,^ ^3^. Although it is an uncommon pathogen^4^, considering its possibility in the absence of a response to initial therapy is crucial. Careful attention to the differential diagnosis of atypical lesions and performing mycobacteriological tests for accurate and timely diagnosis and treatment are necessary^3^ ^,^ ^5^.
